# Metabolomic biomarkers for personalised glucose lowering drugs treatment in type 2 diabetes

**DOI:** 10.1007/s11306-015-0930-4

**Published:** 2016-01-06

**Authors:** Henk den Ouden, Linette Pellis, Guy E. H. M. Rutten, Ilse K. Geerars-van Vonderen, Carina M. Rubingh, Ben van Ommen, Marjan J. van Erk, Joline W. J. Beulens

**Affiliations:** Julius Centre for Health Sciences and Primary Care, University Medical Centre Utrecht, Str. 6.131, PO Box 85500, 3508 GA Utrecht, The Netherlands; TNO, Microbiology and Systems Biology Group, Utrechtseweg 48, 3704 HE Zeist, The Netherlands

**Keywords:** Metformin, Sulphonylurea, Metabolomics, Personalised medicine

## Abstract

We aimed to identify metabolites to predict patients’ response to glucose lowering treatment during the first 5 years after detection of type 2 diabetes. Metabolites were measured by GC–MS in baseline samples from 346 screen-detected type 2 diabetes patients in the ADDITION-NL study. The response to treatment with metformin and/or sulphonylurea (SU) was analysed to identify metabolites predictive of 5 year HbA1c change by multiple regression analysis. Baseline glucose and 1,5 anhydro-glucitol were associated with HbA_1c_ decrease in all medication groups. In patients on SU no other metabolite was associated with HbA1c decrease. A larger set of metabolites was associated with HbA1c change in the metformin and the combination therapy (metformin + SU) groups. These metabolites included metabolites related to liver metabolism, such as 2-hydroxybutanoic acid, 3-hydroxybutanoic acid, 2-hydroxypiperidine and 4-oxoproline). Metabolites involved in oxidative stress and insulin resistance were higher when the HbA1c decrease was larger in the metformin/sulphonylurea group. The associations between baseline metabolites and responsiveness to medication are in line with its mode of action. If these results could be replicated in other populations, the most promising predictive candidates might be tested to assess whether they could enhance personalised treatment.

## Introduction

The management of type 2 diabetes is complex and its complications remain a great burden to individual patients and the larger society (Raz et al. 2013). Incomplete response rates to therapy and the waning durability of response over time with most antidiabetic drugs emphasize the need for personalised interventions to maintain tight glycaemic control (Aquilante 2010). Trial evidence is limited for the optimal use of agents, especially in dual and triple combinations (Raz et al. 2013; Gorter et al. 2012). In clinical practice drugs are prescribed in a trial-and-error manner for each patient to achieve therapeutic targets (Raz et al. 2013). If physicians could predict the patient’s response to treatment, a more individualised approach could be established.

The first line pharmaceutical treatment is metformin. Metformin acts as an insulin sensitizer, suppressing hepatic glucose production and ameliorating insulin resistance in peripheral tissues. In addition, metformin promotes glycogen synthesis and decreases intestinal glucose absorption (Kirpichnikov et al. 2002). Clinical trials showed that metformin has a wide therapeutic response range of HbA_1c_ (glycosylated haemoglobin) reductions from 0.8 to 3 % (Gorter et al. 2012). Moreover, less than two-thirds of patients achieve the fasting glucose target with metformin alone (Hermann et al. 1994). Metformin may be moderately protective against mortality and cardiovascular morbidity (Gorter et al. 2012; Setter et al. 2003).

If needed, mostly sulphonylurea (SU) is added to metformin. Sulphonylureas stimulate insulin release in a glucose-independent manner and may reduce microvascular complications (Inzucchi et al. 2012; DeFronzo 1999). Sulphonylureas lower HbA_1c_ by on average 1–2 % (Gorter et al. 2012; Inzucchi et al. 2012). However, approximately 50–60 % of patients with an initially greater than 30 mg/dl reduction of fasting plasma glucose will fail to reach the desired glycaemic treatment target (DeFronzo 1999). To make patient-centred care and standardized algorithmic management of type 2 diabetes more compatible it is important to know a patient’s responsiveness to treatment (Raz et al. 2013). Thus far, pharmacogenetics have been used to investigate response to glucose lowering treatment with a focus on genetic variations in drug metabolizing enzyme and drug target genes (Pacanowski et al. 2008). Metabolomics is another approach to identify metabolites predicting response to treatment. The advantages of metabolomics over genomics include its direct relation with metabolism and the analysis of relatively few metabolites compared with the unwieldy number of genes. Moreover, metabolomics is more sensitive to detect short-term and/or long-term changes (Lu et al. 2013). During the last decade, metabolomics has provided valuable insights into the pathophysiology of type 2 diabetes (Lu et al. 2013; Bao et al. 2009; Li et al. 2009). Whether metabolomics can be used to investigate response to glucose lowering treatment in screen-detected diabetes patients has not been investigated to date. We aimed to identify metabolic biomarkers to predict patients’ responsiveness to metformin and/or SU during the first 5 years after detection of type 2 diabetes mellitus in a unique population with screen-detected and thus treatment naïve patients with type 2 diabetes mellitus.

## Methods

### Design

This study was performed in the Dutch part of the European ADDITION Study. This randomised, single-blind trial consisted of a screening study and a subsequent intervention study. The practices were randomly assigned to provide routine diabetes care or an intensive multifactorial treatment in a 1:1 ratio by statisticians in each centre according to computer-generated list, independent of measurement teams. The intervention study evaluated the effect of intensified multifactorial treatment on cardiovascular morbidity and mortality in about 3000 screen-detected type 2 diabetes patients aged 40–69 years. Details of the study have been reported previously (Griffin et al. 2011; Van den Donk et al. 2013). For the study website see: http://www.addition.au.dk/. In the ADDITION-Netherlands study 56,978 people aged 50–69 years from 79 primary care practices were invited to participate. Individuals at risk were assessed in general practice and those diagnosed as having type 2 diabetes according to WHO criteria including the requirement for confirmatory testing on a separate occasion, were included in the study. Exclusion criteria were assessed by family physicians. They were illness with a life expectancy of less than 12 months or psychological or psychiatric disorders that might invalidate informed consent, or being housebound or pregnant, or lactation Between 2002 and 2004 586 new type 2 diabetes patients were detected (Janssen et al. 2009). The study was approved by the medical-ethical committee of the University Medical Centre Utrecht. Participants gave written informed consent before study entry.

### Randomisation and interventions

In ADDITION Netherlands 498 screen-detected type 2 diabetes patients were included in a single-blind trial with practice-level randomisation to intensified multifactorial treatment (N = 255) or routine care (N = 243). Allocation was concealed from patients throughout the trial. In total 54 patients were excluded from the longitudinal analyses because they lacked follow-up data. Patients were blinded to which treatment arm their family physician had been randomised.

The patients in the intensive treatment group were treated to achieve an HbA_1c_ <7.0 % (53 mmol/mol). Alternations or additions to glucose-lowering therapy should be initiated when HbA_1c_ >6.5 % (48 mmol/mol). If HbA_1c_ remained above 7.0 % (53 mmol/mol) with oral agents, insulin therapy should be initiated. A healthy diet was advised to all participants (low fat, 600 g of fruit and vegetables/day). (Janssen et al. 2009).

Patients in the routine care group were treated following the guidelines from the Dutch College of General Practitioners. In the 1999 guidelines HbA_1c_ levels between 7.0 % (53 mmol/mol) and 8.5 % (69 mmol/mol) were described as acceptable (Wiersma et al. 1999). In 2006 the HbA1c target became stricter with ≤7.0 % (53 mmol/mol) for all patients (Bouma et al. 2006). Blood pressure and lipid lowering treatments have been described previously (Griffin et al. 2011).

### Measurements

Participants were invited for health assessments at inclusion between 2002 and 2004 and for the final measurement in 2009. If participants did not complete follow-up questionnaires or measurements the most recent values were obtained from the primary care practice records. Between the baseline and final measurement all patients had three-monthly and annual check-ups in the primary care practices. Baseline and subsequent HbA1c and lipid levels were all analysed in one regional laboratory, the SHL Centre for Diagnostic Support in Primary Care, Etten-Leur. HbA1c was analysed with high-performance liquid chromatography using a Menarini 8160 machine. Lipids were determined with standard enzymatic techniques using a Beckman LX-20 until November 2008 and thereafter a Roche Hitachi Modular P. An extra blood sample was taken at baseline and plasma was kept frozen at −80 °C. Participants gave an additional written informed consent for this procedure.

Standardized self-report questionnaires were used to collect information on prescribed medication. Height and weight were measured using a fixed rigid stadiometer and a Tanita scale respectively.

### Metabolomics

Baseline blood samples with sufficient blood volume and without missing study data were defrosted (n = 346). From each sample 100 µl was extracted with methanol and after evaporation the metabolites were derivatized (oximation and silylation). The GC–MS method used for analysing a broad range of metabolites was identical to the method reported for microbial metabolic profiling, (Van der Greef et al. 2007; Wopereis et al. 2009) except for the sample type.

### Performance of the metabolic profiling GC–MS platform

The performance of the applied metabolic profiling platform was assessed through frequent analysis of the Quality Control (QC) sample (Bijlsma et al. 2006). QC samples, prepared from pooled study plasma samples, were analysed after every 10th study sample (in total 72 QC samples). This QC sample represents the full biochemical diversity of the study samples and allows the calculation of the analytical precision for all metabolites measured. The QC sample data is also used to adjust systematic errors (e.g. batch to batch response differences) by a single point calibration model. Typically, this procedure offers excellent precision for a large majority of metabolites (i.e. 77 % of the metabolites have a relative standard deviation (RSD) of less than 10 %). Metabolites with RSD >50 % (very high imprecision), were removed from the data. Furthermore, method performance was carefully monitored using multiple internal standards (5–10 depending on method, including analogues, ^2^H and ^13^C labelled metabolites) and duplicate analysis of samples. Consequently the metabolite data used for statistical data analysis in this study met all of the quality requirements (e.q. RSD < 10 %).

### Pre-processing of metabolic profiling data

Data for each subject were corrected for the recovery of the internal standard for injection. Batch to batch differences in data were removed by synchronizing medians of QC-samples per batch. The GC–MS data set contained 174 metabolites of which 140 were annotated metabolites.

### Statistical analysis

The primary outcome was the relative HbA1c change after 5 years. All values in our analyses were measured at baseline (including all analyses of metabolomics), with the exception of HbA1c after 5 years. Relative HbA1c change was defined as the absolute differences in HbA1c over time adjusted for baseline HbA1c ((HbA_1_c_t5_-HbA_1_c_t0_/HbA_1_c_t0_) × 100 %). So, relative HbA1c change is defined as the absolute differences in HbA1c over time adjusted for baseline HbA1c.

Baseline differences of patient characteristics and all measured metabolites between the medication groups were analysed with ANOVA. To check correlations between all 174 metabolites, Spearman correlations were calculated between all GC-parameters (=GC–MS metabolite) without stratifying for medication groups (n = 346). A mixed model was made per GC parameter with the relative change in HbA_1c_ as dependent factor in the model and the continuous GC parameter (measured at baseline) as an independent variable in the model. Medication group was included as an independent variable as well and included as a fixed factor. Finally, the interaction of GC parameter with medication group was included as an independent variable in the model. In this analysis, the no medication group was used as the reference group for the interaction between GC parameter and medication. The beta for the interaction of the GC parameter with that medication group is reported here for each medication group. This beta represents the additional contribution of each metabolite in the specific medication group compared with the no medication group. The model was run with data from all subjects as well as with data from the subset of subjects with HbA_1c_ >6.5 % at start of the study (n = 219). This level was the threshold to start oral blood glucose lowering therapy and is nowadays used as threshold for the diagnosis of diabetes (Grundy 2012). In a secondary analysis, the results were adjusted for baseline BMI and baseline HbA1c, since these parameters were significantly different between the medication groups at baseline. Multiple testing correction was performed by submitting the data to Benjamini and Hochberg test (Benjamini and Hochberg 1995). Statistical analyses were done with SAS version 9.3.

## Results

Patients (n = 346) were divided into groups according to use of medication after 5 years of follow-up: no medication (n = 82), only metformin (n = 132), the combination metformin and SU (n = 94), and only SU (n = 38). The four groups were comparable at baseline with respect to age and blood pressure, but baseline HbA_1c_, body weight, BMI, waist circumference, and cholesterol levels differed significantly between the groups (Table 1). In patients who were prescribed combination therapy HbA1c differed significantly from both other groups: 8.2 % (66 mmol/mol) versus 7.3 % (56 mmol/mol) (metformin) and 7.0 % (53 mmol/mol) (SU). Patients who were prescribed combination therapy differed significantly in weight from those on metformin alone (88.2 and 93.8 kg respectively). The baseline BMI of patients on metformin alone differed significantly from the BMI in the other groups. Of all metabolites, 22 (12.6 %) of all measured metabolites were significantly different at baseline between medication groups. Of these metabolites, five showed a significant interaction with medication group on relative HbA1c change (oxoproline, hydroxypiperidine, uric acid, glutamic acid internal amide (formed during derivatisation step, measure for glutamate), and pseudouridine).

**Table 1:** Baseline characteristics of the different medication groups

	No med (n = 82)	Metf (n = 132)	SU (n = 38)	Combi (n = 94)	All (n = 346)
	Mean (SD)	Mean (SD)	Mean (SD)	Mean (SD)	Mean (SD)
Age (years)	60.7 (5.1)	59.6 (5.2)	60.6 (6.0)	60.0 (5.2)	60.1 (5.3)
SBP (mmHg)	169.7 (20.7)	162.7 (20.2)	162.5 (27.6)	162.4 (26.3)	164.3 (23.1)
DBP (mmHg)	86.9 (7.3)	89.9 (9.9)	93.1 (11.1)	88.0 (11.9)	89.3 (10.7)
Cholesterol (mmol/l)*	5.6 (1.0)	5.6 (1.0)	5.8 (1.3)	5.7 (1.1)	5.6 (1.1)
LDL (mmol/l)	3.5 (0.9)	3.7 (0.9)	3.9 (1.2)	3.8 (0.9)	3.7 (1.0)
HbA_1c_ (%)*	6.3 (0.8)	7.3 (1.4)	7.0 (1.1)	8.2 (1.8)	7.3 (1.5)
BMI (kg/m^2^)*	29.2 (4.3)	31.9 (4.7)	29.7 (4.3)	30.3 (4.4)	30.6 (4.6)
Weight (kg)*	85.9 (15.7)	93.8 (15.6)	86.1 (16.7)	88.2 (15.1)	89.5 (15.9)
Waist circumference*	104.2 (12.2)	110.0 (11.4)	104.6 (14.6)	106.6 (11.7)	107.1 (12.2)
Statin use (n, (%))	7 (8.8)	17 (13.2)	5 (14.3)	14 (15.1)	43 (13.1)

*No Med* no medication, *Metf* metformin, *SU* sulphonylurea, *Combi* combination of metformin and sulphonylurea, *SBP* systolic blood pressure, *Cholesterol* total cholesterol, *LDL* low-density lipoprotein cholesterol,* HbA*_1c_ glycated haemoglobin, *BMI* body mass index

* Groups differ significantly (p < 0.05)

**Fig. 1:**
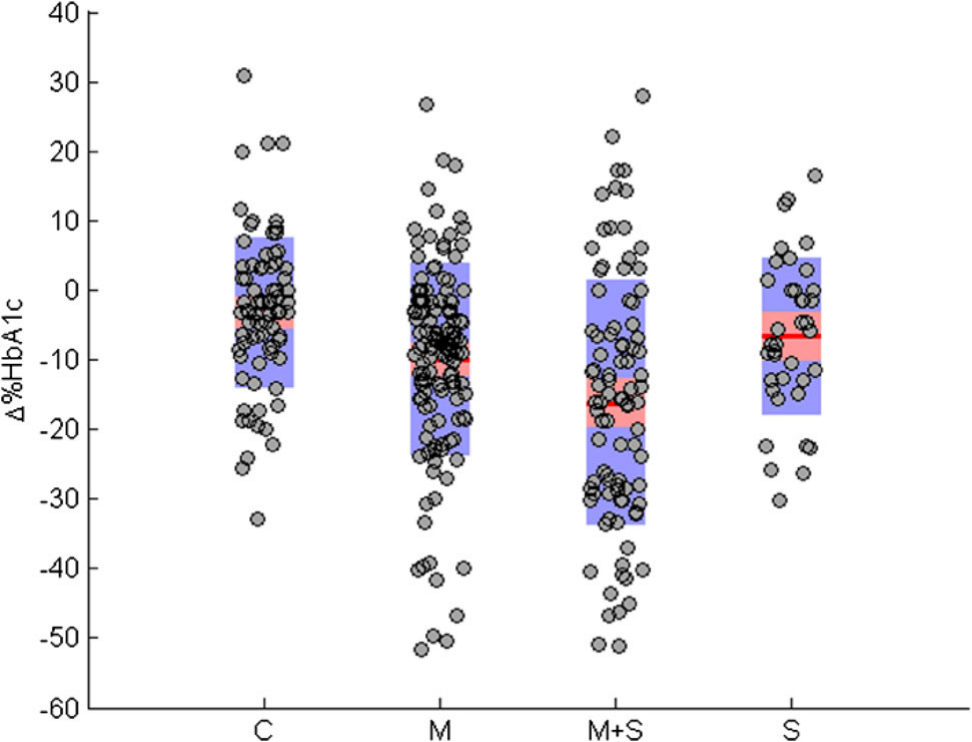
Relative HbA_1c_ after 5 years for each medication group (∆ %HbA_1c_ = ((t5-t0/t0)*100 %)) (C = no medication, M = metformin, M + S = combination metformin and sulphonylurea, S = sulphonylurea, *red* mean, *pink* 1 SD, *blue* 95 % confidence interval and *ash* individual data), n = 264 (Color figure online)

Figure 1 shows a large variation in response to glucose lowering drug treatment after 5 years. The metformin and SU combination group showed both the largest decrease and variation in 5 year change of HbA_1c_ with a mean of −16.3 mmol/mol and a range of −28.7 to −6.0 mmol/mol, while the control group (no medication) had the smallest decrease and variation in 5 year HbA_1c_ change with a mean of −3.2 mmol/mol and range −8.1 to 3.1 mmol/mol.

Spearman correlations between all 174 metabolites (30.102 in total) were generally low with only 5.8 % of coefficients above 0.4, of which a majority ranked between 0.4 and 0.6.

Using spearman univariate analyses among all subjects, only 1.5 anhydro-glucitol (0.537) and glucose (−0.419) were significantly correlated with 5 year change in HbA_1c_. Only these associations remained significant after adjusting for multiple testing (FDR corrected *p* value < 0.05). No correlations were found between age, weight, BMI and waist circumference and relative HbA1c change in the entire study population (data not shown).

Table 2 shows the baseline metabolite values with an unadjusted significant interaction with medication group on relative HbA_1c_ change after 5 years in the three groups.

**Table 2:** Metabolites with a significant unadjusted interaction with medication group on relative HbA_1c_ change in the entire study population (n = 264)

	Metformin		Sulphonylurea (SU)		Metformin and SU	
	Coefficient	p value	Coefficient	p value	Coefficient	p value
1,5 anhydroglucitol (HMDB 02712, CAS 154-58-5)	14.2	0.001	10.8	0.043	29.8	<0.0001
2-hydroxybutanoic acid (HMDB 00008, CAS 5094-24-6)	−60.7	0.011	14.6	0.716	−68.5	0.013
2-hydroxypiperidine (Pubchem 24847875, CAS 5382-16-1)	781.4	0.016	333.5	0.511	1164.2	0.002
3-hydroxybutanoic acid (HMDB 00357, CAS 300-85-6)	−18.2	0.029	−4.6	0.850	−54.3	0.015
4-oxoproline (KEGG C01877, CAS 4347-18-6)	517.9	0.002	409.5	0.096	682.0	0.001
Glucose (HMDB 00122, CAS 50-99-7)	−1.7	0.001	−1.3	0.043	−1.8	0.0003
Glutamic acid internal amide^a^ (HMDB 00267, CAS 98-79-3)	26.6	<0.0001	9.8	0.296	15.6	0.026
Myo-inositol (HMDB 00211, CAS 87-89-8)	51.3	0.050	6.7	0.818	106.0	0.038
Pseudo uridine (HMDB 00767, CAS 1445-07-4)	109.3	0.012	35.1	0.519	140.1	0.007
LCB 18:1-17:0 SM	1079.0	0.044	409.7	0.586	1830.9	0.003
l-Methionine (HMDB 00696, CAS 63-68-3)	191.1	0.018	101.8	0.363	25.1	0.768
l-Phenylalanine (HMDB 00159, CAS 63-91-2)	24.3	0.034	17.0	0.317	2.5	0.843
4-Hydroxyglutamate semialdehyde (HMDB 06556)	654.1	0.034	527.3	0.192	457.6	0.162
LCB18:0-16:0 SM (HMDB 10168)	30.2	0.334	33.3	0.500	93.5	0.010
LCB18:1-18:0 SM (HMDB 01348, CAS 58909-84-5)	−0.9	0.763	4.5	0.236	6.6	0.038
Uric acid (HMDB 00289, CAS 69-93-2)	0.7	0.163	0.4	0.526	1.3	0.020

This beta represents the additional contribution of each metabolite in the specific medication group compared with the no medication group

^a^Formed during derivatisation step, measure for glutamate

In the metformin group, high levels of 3-hydroxybutanoic acid and low levels of 2-hydroxypiperidine and 4-oxoproline were associated with the 5 year HbA_1c_ change. In the combined therapy group, similar associated metabolites were identified. All above mentioned correlations became stronger in the combination group. Other significant metabolites in the metformin group are glutamic acid internal amide, myo-inositol, pseudo uridine, LCB 18:1–17:0 SM, l-methionine, l-phenylalanine, 4-hydroxyglutamate hydroxyaldehyde and 2-hydroxybutanoic acid. Furthermore, lower concentrations of sphingomyelins (18:0–16:0, 18:1–18:0, 18:1–17:0), pseudo uridine, myo-inositol, glutamic acid internal amide and uric acid baseline were associated with a larger decrease in HbA_1c_ in the combination group.

In patients who were prescribed only SU, no other metabolite was correlated with the decrease in HbA_1c_ after 5 years besides glucose and 1.5 anhydroglucitol.

Adjusting for baseline differences in BMI did not substantially alter our results in all groups (data not shown). However, after adjusting for baseline differences in HbA_1c_ and BMI in all groups, only 1,5 anhydroglucitol (p < 0.033), 2-hydroxybutanoic acid (p < 0.003), 2-hydroxypiperidine (p < 0.012), glucose (p < 0.029), sphingomyelin 18:1-17:0 (p < 0.040) and phenylalanine (p < 0.048) remained significant.

When restricting to 219 patients with an HbA_1c_ > 6.5 % at start of the study (Table 3), we generally observed comparable results. Although the metabolites are different, the metabolites are involved in the same biological processes. Regardless of medication groups, 1.5 anhydro-glucitol and glucose, glutamic acid internal amide and 4-hydroxy hydroxyglutamate semialdehyde were associated with the 5 year change in HbA_1c_. In the metformin group, higher levels of 2-hydroxybutanoic acid, 3-hydroxybutanoic acid and 3-amino-2-piperidon and lower levels of 2-hydroxypiperidine and 4-oxoproline were associated with a larger decrease in HbA_1c_. In the combined therapy group, similar metabolites were identified with mostly stronger associations. Furthermore, in the combined therapy group lower levels of two sphingomyelins (18:0-16:0 and 18:1-17:0) and myo-inositol were associated with a larger 5 year HbA1c decrease, as well as higher baseline levels of four fatty acids (C14:0, C17:0, C18:0, C20:1), mannose and xanthine. In the SU group, high levels of fumaric acid were associated with a greater decrease in HbA_1c_ after 5 years.

**Table 3:** Metabolites with a significant unadjusted interaction with medication group on relative HbA_1c_ change among patients with HbA_1c_ > 6.5 %

	Metformin		Sulphonylurea (SU)		Metformin and SU	
	Coefficient	p-value	Coefficient	p-value	Coefficient	p-value
Glucose (HMDB 00122, CAS 50-99-7)	−2.7	0.0003	−2.2	0.012	−2.8	0.0002
Glutamic acid internal amide (HMDB 00267, CAS 98-79-3)	39.1	0.001	28.1	0.042	29.5	0.009
1,5 anhydroglucitol (HMDB 02712, CAS 154-58-5)	23.7	0.003	21.2	0.018	42.4	<0.0001
4-hydroxyglutamate semialdehyde (HMDB 06556)	1327.7	0.007	1219.7	0.030	1180.1	0.018
2-hydroxybutanoic acid (HMDB 00008, CAS 5094-24-6)	−85.8	0.009	−6.5	0.898	−110.6	0.002
3-amino 2 piperinidon (HMDB 00323, CAS 1892-22-4)	−23.2	0.013	−250.8	0.792	−1535.5	0.023
3 hydroxybutanoic acid (HMDB 00357, CAS 300-85-6)	−23.2	0.013	−39.4	0.297	−58.3	0.012
4-oxoproline (KEGG C01877, CAS 4347-18-6)	464.6	0.029	224.7	0.477	576.8	0.022
2-hydroxypiperidine (Pubchem 24847875, CAS 5382-16-1)	1126.4	0.045	696.3	0.319	1283.3	0.033
Xanthine (HMDB 00292, CAS 69-89-6)	2086.6	0.501	−4215.6	0.261	−8405.6	0.004
C20:1 fatty acid (HMDB 02231, CAS 26764-41-0)	−1590.6	0.103	−1724.4	0.176	−2701.4	0.009
C14:0 fatty acid (HMDB 00806, CAS 544-63-8)	−267.3	0.243	−219.4	0.534	−527.4	0.026
C18:0 fatty acid (HMDB 00827, CAS 57-11-4)	−804	0.231	−27.1	0.771	−145.8	0.030
C17:0 fatty acid (HMDB 02259, CAS 506-12-7)	−1273.0	0.476	368.5	0.880	−3742.5	0.039
Mannose (HMDB 00169, CAS 3458-28-4)	−58.5	0.425	−13.2	0.898	−152.6	0.042
LCB 18:1-17:0 SM	1348.7	0.090	675.3	0.493	1702.6	0.043
Myo-inositol (HMDB 00211, CAS 87-89-8)	53.1	0.083	−12.2	0.735	67.2	0.044
LCB 18:0-16:0 SM (HMDB 10168)	61.8	0.217	67.8	0.351	105.4	0.046
Fumaric acid (HMDB 00134, CAS 110-17-8)	−979.8	0.266	−2234.0	0.044	−926.0	0.329

## Discussion

This study shows a large variation in response to glucose lowering drug treatments in screen detected type 2 diabetes patients. In the different treatment groups, different metabolites could be identified that were associated with the response to metformin and/or sulphonylureas. This indicates that metabolomics can be used as a tool to identify potential biomarkers for response to diabetes treatment.

Regardless of medication, high plasma levels of glucose and low plasma 1,5-anhydroglucitol at the time of screen-detection were associated with the HbA_1c_ decrease after 5 years. Only these markers remained significant after adjustment for multiple testing. The metabolite 1,5 anhydro-glucitol is a well-known short term biomarker of hyperglycaemia (48 h–2 weeks). As a result of glucose’s competitive inhibition of 1,5-anhydroglucitol reabsorption in the kidney tubule, these concentrations are low during hyperglycaemia (Lyons and Basu 2012; Pal et al. 2010; McGill et al. 2004). As expected, our results show that subjects with a larger dysregulation in glucose metabolism were more prone to respond to glucose lowering treatment regardless of medication and BMI. In line with previous studies we found that the predictive values of other characteristics such as age, BMI and lipid levels at baseline are small in predicting the change in HbA_1c_ after follow-up (Prentki and Madiraju 2012; Goudswaard et al. 2004; Janghorbani and Amini 2012).

In patients on metformin, high levels of liver metabolites 2-hydroxybutanoic acid and 3-hydroxybutanoic acid at diagnosis were correlated with a larger decrease in HbA_1c_ after 5 years. Hydroxybutanoic acid is produced mainly in the liver, during detoxification or oxidative stress (Brosnan and Brosnan 2009; Wu et al. 2004). 3-Hydroxybutanoic acid is a ketone body that decreases after stimulation of the glucose metabolism (Shaham et al. 2008). Metformin usage increases serum 3-hydroxybutanoic acid levels in type 2 diabetes (Huo et al. 2009). Likewise 2-hydroxybutanoic acid is an early biomarker of insulin resistance in non-diabetic subjects and increased in diabetes type 2 patients (Gall et al. 2010; Li et al. 2009). One could hypothesize that subjects with high levels of these liver metabolites might have insulin resistance in the liver (DeFronzo 2009). Also 4-oxoproline was identified as a metabolite to predict response to metformin. Oxoproline is an intermediate in arginine and proline metabolism, which can be used for glutamate production and forms a link between the tricarboxylic acid and urea cycle (Bertolo and Burrin 2008). Both 2-hydroxybutanoic acid and oxoproline indicate an increased liver metabolism, in line with the mode of action of metformin that specifically acts on the liver by blocking hepatic gluconeogenesis (Gallagher and LeRoith 2011). One could postulate that type 2 diabetes patients with glucose dysregulation and increased liver metabolism will respond well to metformin treatment. This is in line with the results in the metformin and sulphonylurea combination group, where high plasma levels of liver metabolites 2-hydroxybutanoic acid, 3-hydroxybutanoic acid, and low levels of 2-hydroxypiperidine, 4-oxoproline were also correlated with a larger decrease in HbA_1c_ after 5 years.

In the metformin/SU combination group, we could also identify mannose, xanthine and uric acid as metabolites associated with HbA_1c_ change. Oxidative stress is increased in type 2 diabetes compared to healthy subjects and corresponding metabolites like mannose and uric acid are increased with oxidative stress (Gall et al. 2010; Suhre et al. 2010). Xanthine oxidase is also increased in oxidative stress and is an enzyme involved in uric acid synthesis (Dikalov 2011). Low myo-inositol concentrations were also associated with a higher decrease in HbA_1c_ after 5 years. Indeed, myo-inositol concentrations are lower in insulin resistant subjects (Gall et al. 2010). Myo-inositol is involved in the activation of protein kinase C (PKC), which plays an important role in glucose metabolism (Nishizuka 1995; Lamb and Goldstein 2008).

In addition, four fatty acids were found to be higher at baseline in subjects that had the largest decrease in HbA_1c_, receiving both metformin and sulphonylurea. Free fatty acids originate from adipose tissue (Prentki and Madiraju 2012; Capurso and Capurso 2012) and may cause insulin resistance (Capurso and Capurso 2012). It is known that insulin resistance and increased oxidative stress can be caused by multiple organs dysregulation.

Increased C18:0 is found in serum of type 2 diabetics (Kellow et al. 2011). Impaired glucose tolerant subjects have increased C14:0, C17:0 and C18:0 fatty acids levels (Gall et al. 2010) and C14:0, C17:0, C18:0 and C20:1 levels are increased in diabetics compared to insulin sensitive subjects (Suhre et al. 2010). Altogether, we have identified several metabolites involved in insulin resistance in adipose tissue. This could indicate that when subjects have adipose tissue insulin resistance in addition to liver insulin resistance, they should be placed on combination therapy.

Altogether, one could postulate that subjects with glucose dysregulation in multiple organs (liver and adipose tissue) would better respond to a combined metformin/sulphonylurea treatment.

In the SU group only high levels of fumaric acid were correlated to decrease in HbA_1c_ after 5 years, but only in subjects with HbA_1c_ over 6.5 % at baseline. Fumarate is involved in the tricarboxylic acid cycle, necessary for the insulin secretion by the ß-cells of the pancreas (Bain et al. 2009). Sulphonylureas stimulate insulin release in a glucose-independent manner by acting on the ß-cells of the pancreas. One could postulate that subjects with glucose dysregulation and altered pancreatic metabolism would better respond when prescribed SU treatment.

Specifically in subjects with an HbA_1c_ above 6.5 % at baseline, low glutamic internal amide (as a marker of glutamate) and 4-hydroxy glutamate semialdehyde were associated with the decrease in HbA_1c_ after 5 years in all medication groups. Elevated blood levels of the former may be associated with problems of glutamine or glutathione metabolism. (Brosnan and Brosnan 2009; Brosnan 2000). 4-Hydroxyglutamate semialdehyde is an intermediate in arginine and proline metabolism, which can be used for glutamate production (Brosnan 2000). Glutamate plays a central role in hepatic amino acid metabolism, maintaining normal amino acids concentrations and energy usage (Brosnan and Brosnan 2009; DeFronzo 2009). Plasma glutamate levels are elevated in several diseases characterized by chronic oxidative stress and inflammation, like obesity and type 2 diabetes (Davalli et al. 2012). Since low levels of both these glutamate related metabolites were associated with HbA_1c_ decrease, one could hypothesize that our data indicate that drug treatment could still be effective since our subjects were newly diagnosed and therefore the glutamate-induced cytotoxicity (Davalli et al. 2012) had not yet taken place.

Strengths of this study include the quite large patient group of screen detected diabetes patients before use of any antidiabetic drug and the long follow-up time with a median of approximately 6 years. However, certain limitations need to be addressed. The number of patients per group differs from 38 to 132 which makes some analyses less robust. This difference was due to the ADDITION-treatment algorithm that suggested to start with metformin, to add a SU if necessary and to treat a patient with SU monotherapy in case of contra-indications for or side effects of metformin (Griffin et al. 2011). Although the metabolites identified in this study are all well-known metabolites associated with oxidative stress, insulin resistance or type 2 diabetes, our results should be seen as hypothesis generating and require further investigation. Because of multiple testing, our results are prone to false positive findings. Indeed, when we adjusted our p-values for multiple testing, only metabolites of dysregulation remained significant. This is probably due to the relatively small sample size of this study. This also makes it difficult to predict which of the other markers are least likely to be false positives. Although we identified metabolites that are biologically plausible to predict response to the different hypoglycaemic treatments, these results need to be replicated in independent populations.

We observed that total cholesterol levels, but not LDL cholesterol levels, were different between the medication groups. Therefore we checked statin use between our defined medication groups. Importantly, it was not different, since the use of statins increases the risk of elevation of blood glucose (Chapman et al. 2011). The use of blood pressure lowering drugs could have been different between the three glucose lowering medication groups and influence the outcome, but was not analysed.

Furthermore, we were certain of the use of medication in the population, but the dosage and duration of the SU and metformin use during the follow up period of 6 years are uncertain. Of all participants to the ADDITION-study 96 % was of Caucasian race. So race is of minimal influence on the results presented in our study. Finally, when the results were adjusted for baseline HbA_1c_ several metabolites lost significance. This indicates that certain metabolites were driven by baseline HbA_1c_ levels. However, our results show that not only baseline HbA1c determines 5 years HbA1c change. Moreover, perhaps the metabolites that were independent from baseline HbA1c could be regarded as the most promising ones for further investigation.

In conclusion, we aimed to identify metabolites to predict response to metformin and/or SU treatment during 5 years after detection of type 2 diabetes. Apart from markers of glucose dysregulation, we identified metabolites associated with 5 year HbA_1c_ change that were in line with the mode of action of metformin, sulphonylureas or the combination therapy. If these results could be replicated in other populations, the most promising predictive candidates might be tested to assess whether they could enhance personalised treatment.
